# Active Verification for Missing-Annotation-Aware Tiny Surface Defect Detection in Resistors

**DOI:** 10.3390/s26123912

**Published:** 2026-06-19

**Authors:** Chengdi Zhang, Mingxuan Yu, Wenzhang Dong, Jiaxuan Zhan, Shengdong Yu, Jinyu Ma, Mingyang Xie

**Affiliations:** 1 School of Mechanical and Electrical Engineering, Wenzhou University, Wenzhou 325000, China; chengdizhang@stu.wzu.edu.cn (C.Z.); jiaxuanzhan@stu.wzu.edu.cn (J.Z.); shengdong@nuaa.edu.cn (S.Y.); 2 Yikun Electric Co., Ltd., Wenzhou 325000, China; mingxuanyu@aliyun.com (M.Y.); ykblq@yikun.cn (W.D.); 3 College of Automation Engineering, Nanjing University of Aeronautics and Astronautics, Nanjing 210016, China; 4 School of Intelligent Manufacturing, Wenzhou Polytechnic, Wenzhou 325000, China

**Keywords:** resistor surface inspection, missing annotation, active verification, label repair, prototype consistency, tiny defect detection

## Abstract

In the resistor images used in this study, many defective regions are weak coating-like marks rather than obvious scratches or pits. Their appearance is close to the epoxy background, and some visible defects were missing from the original annotation files. If these labels are used directly, the detector treats the missed defects as background samples during training. We therefore corrected the supervision before changing the feature constraint. An early YOLO26s model was first used to nominate low-overlap boxes, and these candidates were then checked manually. Only confirmed defects were merged into the labels. After this step, a scale-gated prototype consistency term was added during training to reduce the model’s bias toward the dominant tiny-defect group. On the fixed corrected benchmark, mAP50 improved from 28.14% to 63.20%, and Recall increased from 18.42% to 62.20%. In the end-to-end deployment view, where the raw and cleaned validation sets answer different practical questions, mAP50 changed from 43.66% to 63.15%, and Recall changed from 30.01% to 62.24%. For normal-size defects, Recall increased from 26.09% to 56.52%. A prototype-only transfer study on the public MVTec AD benchmark further evaluates whether the feature constraint generalizes when the label-repair stage is not applicable to clean public annotations. Since the prototype term is removed after training, the deployed detector remains the original YOLO26s model without an additional inference branch.

## 1. Introduction

The resistor samples in this work have epoxy-coated surfaces. In the collected images, the defects are often not sharp cracks, pits, or stains [1]. Many of them appear as shallow coating marks [2], and their color is very close to the surrounding epoxy. This makes the inspection problem less like detecting a clear object and more like separating a weak local abnormality from normal surface variation [3].

The difficulty becomes greater after the image enters a deep detector. Some annotated boxes cover only a few pixels, so their responses can be weakened after several downsampling operations [4]. A larger input size [5] or shallow-feature fusion [6] may preserve more detail, but these changes alone do not solve the confusion between a true defect and normal coating texture.

During label checking, we found that several visible defect regions had no corresponding boxes. This is more serious than ordinary annotation noise in the present task [7]. An unlabeled defect is used as a background sample during training [8], although visually it belongs to the defect class. As a result, the detector receives contradictory supervision around the defect-background boundary [9].

Most existing tiny-defect methods try to recover weak signals by changing the network, for example by using a stronger backbone [10], an attention block [11,12], or a multi-scale fusion module [13,14]. These designs are useful, but they assume that the training target is basically correct. In the present dataset, this assumption is not safe. If a real defect is missing from the label file, a better feature extractor may still be optimized toward an incorrect background target [15].

Even when the missing-label issue is handled, a second challenge remains. In the surface defect detection, there are some defect regions whose size varies significantly across samples, and the annotating of such regions is easily affected by the uneven target-size distribution. Industrial defect datasets commonly exhibit a long-tailed size distribution: extremely small flaws dominate the training samples, while normal-size or morphologically atypical defects are rare. A detector trained under such imbalance tends to over-specialize its feature representations on the dominant tiny scale, delivering erratic predictions when encountering non-dominant sizes [16].

In few-shot learning, prototypical networks learn a metric space in which classification is performed by computing distances to prototype representations of each class [17]. Prototype distillation extends this idea to object detection [18], and prototype-guided anomaly localization applies it to industrial anomaly detection [19,20], anchoring more stable class centers and tightening intra-class feature cohesion. Those ideas inform the feature-level component developed in the present study.

Recent studies on noisy-label learning, prototype-guided industrial anomaly detection, and active learning for surface inspection show that label quality and representation geometry are often intertwined rather than independent issues. However, most network-level tiny-defect detectors assume that the target boxes are already reliable, while most noisy-label methods re-weight, select, or soften existing annotations without physically recovering defect instances that are absent from the label file. We therefore separate the claim levels: the corrected resistor benchmark supports the complete pipeline, while MVTec AD supports the prototype term alone. Within the MVTec detection study, the comparison focuses on the controlled YOLO-family setting, because the purpose is to evaluate whether the prototype constraint transfers when added to the same baseline family.

Based on these observations, this work treats label quality as the first issue to be addressed. The proposed framework first repairs missing annotations through active verification [21] and then applies a prototype consistency constraint to reduce size-related feature bias. On the data side, a human-in-the-loop verification loop finds and fixes labels that are missing in both training and validation sets. A baseline detector flags suspicious regions; a human reviewer checks each candidate, and only confirmed boxes are merged into the label files. On the feature side, defect-class prototypes are constructed in the learned feature space, and a consistency penalty is applied to positive samples that pass an area-gating check. The aim is not to build a heavier detector, but to improve the supervision and feature geometry of a lightweight model used in production-line inspection.

The main contributions of this work are threefold. First, a human-in-the-loop active verification method is designed to address missing labels in industrial defect data by chaining model-generated nominations with binary human confirmation and merging the corrected annotations, thereby repairing both training and validation sets at low cost; cleaner training labels provide a more accurate optimization target, while corrected validation labels prevent real defects from being incorrectly penalized as false positives. Unlike conventional image-level active learning, the proposed verification loop operates at the box level. A candidate is nominated only when the detector predicts a region with low overlap to existing annotations, and the human action is a binary confirmation rather than full relabeling. Second, after supervision repair, a scale-aware prototype consistency constraint is introduced to mitigate feature bias under long-tailed size distributions [16] by constructing defect-class prototypes in the feature space and applying an area-gated consistency penalty to qualifying positive samples, thereby stabilizing the representations of less frequent defect sizes. The two stages are intentionally ordered: applying prototype consistency before label repair would construct the prototype from contaminated supervision, whereas repairing labels first provides a more trustworthy anchor for feature regularization. Third, the inference model is not enlarged. The prototype term is calculated only when the training loss is formed. After training, the deployed model is still YOLO26s, without an added prototype branch, extra feature comparison, or additional post-processing step.

The paper is organized as follows. Section 2 gives the proposed method. Section 3 presents the experimental setup and results. Section 4 concludes the paper.

## 2. Proposed Method

### 2.1. Problem Definition and Overall Framework

The images used in this study come from epoxy-coated resistor surfaces. Most defects are small local marks related to coating variation, curing differences, or handling contact [22]. In many samples, the defect boundary is not clear, and the local color change is close to the normal background. This weak visual contrast makes both manual labeling and detector training sensitive to small differences in texture.

To clarify the dataset composition, the cleaned validation benchmark contains 58 images and 975 confirmed defect instances. The size-distribution analysis uses 3193 confirmed boxes after label repair: 2438 tiny boxes (76.35%), 634 small boxes (19.86%), and 121 normal-size boxes (3.79%). No separate held-out test set is available in the present production dataset; this limitation is stated explicitly. The detailed dataset composition is summarized in Table 1.

When these images are processed by YOLO26s, the weak defect response may be further reduced in the feature hierarchy [23]. Tiny boxes are especially affected because only a small number of pixels contribute to the corresponding feature. Batch-to-batch changes in coating condition also change the background texture [24]. Therefore, if the labels are incomplete, the model is likely to learn an unstable defect-background boundary.

The annotation condition is equally important for this task. The performance of deep learning-based methods is heavily dependent on the availability of accurately labeled datasets, but incomplete labels are common in industrial datasets. Training a detector directly on such annotations causes real but unlabeled defects to be optimized as background. This behavior damages the defect-background boundary and reduces the model’s ability to generalize. For this reason, the proposed framework treats label repair as the first step rather than as an optional preprocessing operation. After the supervision boundary is corrected by active verification, a prototype consistency constraint is introduced to regularize the feature space and reduce the scale bias caused by the long-tailed size distribution. The framework is therefore organized into two connected stages. Stage I repairs supervision boundaries through human-in-the-loop active verification; Stage II applies a scale-aware prototype consistency constraint on the repaired labels to improve the response to non-dominant defect sizes [25].

**Figure 1:**
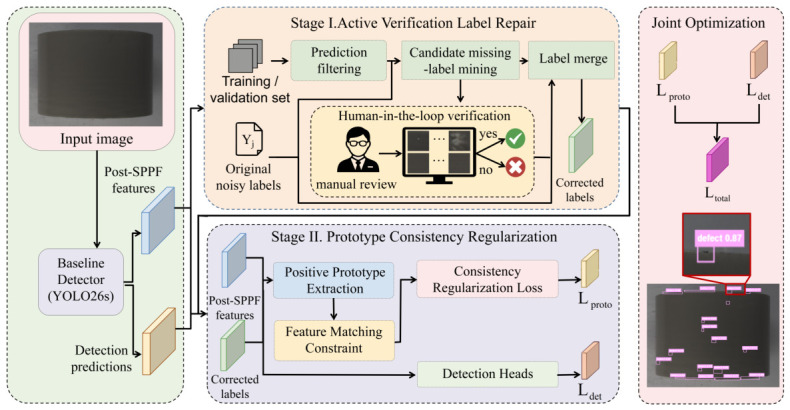
Overall framework of the proposed method.

The complete workflow is illustrated in Figure 1. The input image is first fed into the baseline detector to obtain detection predictions and intermediate-layer features. Then, Stage I exploits the discrepancy between model predictions and original annotations to mine potential missing-label candidates and completes label repair through a human-in-the-loop active verification mechanism. On this basis, Stage II further constructs a prototype consistency constraint using the corrected labels and deep features, imposing smooth regularization on the feature space under long-tailed size distributions. Finally, the detection loss and prototype consistency loss are jointly optimized to drive model parameter updates.

The right side of Figure 1 shows the loss used in the second training stage. The normal YOLO26s detection loss is kept, and the prototype term is added only when the training loss is calculated. The prototype operation is not used during testing. Therefore, the exported detector still follows the original YOLO26s inference path, without an additional prototype branch, feature comparison module, or post-processing step. In our pipeline, label repair is performed first to correct the missing defect boxes. The repaired labels are then used for feature regularization, so that the model is less dominated by the numerous tiny defects and can better respond to less frequent defect sizes.

### 2.2. Baseline Detection Model and Loss Formulation

**Figure 2:**
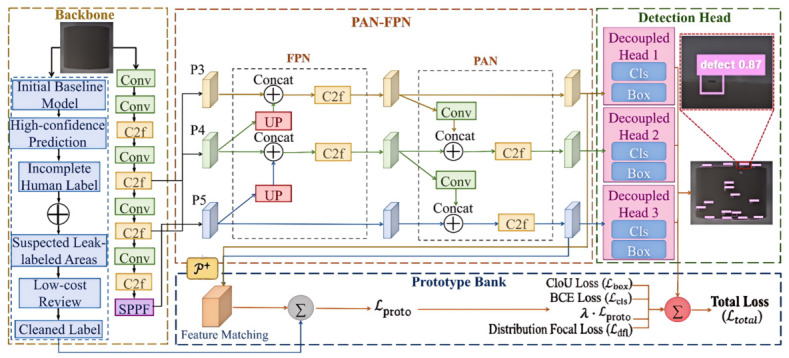
Overall architecture of the proposed defect detection method.

Considering the industrial production line requirements for inference latency, parameter scale, and deployment stability, this paper selects the YOLO26s one-stage detector as the baseline architecture. YOLO26s denotes the small-scale YOLO26 configuration used in the Ultralytics-style implementation; the model used here has 9.47M parameters and 20.5 GFLOPs. The configuration file and training hyperparameters are provided with the revised experimental protocol to support reproducibility. The proposed detection framework is shown in Figure 2. The framework takes active-verification-based label repair as the main line and introduces a prototype consistency constraint under corrected supervision to mitigate scale bias. Given an input image, the detection model performs backbone feature extraction and cross-scale fusion through the feature pyramid, and then outputs a set of candidate bounding boxes:

(1) $$D\left(I\right)={\left\{\left(b_{i},s_{i},c_{i}\right)\right\}}_{i=1}^{N}$$

where $b_{i}$ denotes the coordinate regression parameters of the *i*-th predicted box, $s_{i}$ denotes the corresponding object confidence score, $c_{i}$ denotes the predicted class label, and *N* denotes the total number of candidate boxes output by the model for the current image.

During training, this paper adopts a combined optimization form consisting of bounding-box regression loss [26], classification loss, and distribution focal loss [27]. The overall detection loss is defined as:

(2) $$L_{det}=\lambda_{box}L_{CIoU}+\lambda_{cls}L_{BCE}+\lambda_{dfl}L_{DFL}$$

where $L_{CIoU}$ constrains the geometric consistency between predicted boxes and ground-truth boxes; $L_{BCE}$ is the binary cross-entropy classification loss; and $L_{DFL}$ is the distribution focal loss. $\lambda_{box}$, $\lambda_{cls}$, and $\lambda_{dfl}$ denote the weight coefficients of the three loss terms, respectively.

In industrial tiny defect detection, defects are usually small, boundary-blurred, and weakly textured. Let *y* denote a coordinate value of the ground-truth bounding box falling within the predefined discrete interval $\left[y_{i},y_{i+1}\right]$. The distribution focal loss can be formulated as:

(3) $$L_{DFL}=-\left[\left(y_{i+1}-y\right)\log P\left(y_{i}\right)+\left(y-y_{i}\right)\log P\left(y_{i+1}\right)\right]$$

This loss assigns continuous interpolation weights to adjacent discrete positions, enabling the model to represent boundary locations in the form of probability distributions and thereby enhancing regression stability for weak-boundary targets.

The above detection losses assume that supervision labels are complete and reliable. In real industrial scenarios, however, training data often contain missing-label noise. The classification loss then incorrectly suppresses unlabeled real defect regions as background, which interferes with the joint optimization of classification and regression. For this reason, it is necessary to repair missing-label supervision at the data level before introducing additional feature constraints.

### 2.3. Active-Verification-Based Label Repair Mechanism

As noted in surveys on learning from noisy labels, noisy labels severely degrade the generalization performance of deep neural networks. To address the feature-boundary confusion caused by missing-label supervision, this paper proposes an active verification label repair mechanism driven by model predictions. The core idea is to exploit the early training stage of the baseline detector, before it has fully overfitted noisy background regions, to actively mine potential missing-label regions from prediction results. These regions are then corrected through low-cost human-machine collaborative verification, thereby restoring more faithful supervision boundaries [28].

Let the noisy-labeled training set be $T={\left\{\left(I_{j},Y_{j}\right)\right\}}_{j=1}^{M}$. A pretrained baseline model $D_{0}$ is used to perform forward inference, producing a predicted box set ${\hat{Y}}_{j}$. If the maximum IoU between a predicted box $\hat{b}$ and any ground-truth box *b* in $Y_{j}$ is smaller than a threshold τ, the predicted box is included in the suspected missing-label candidate set:

(4) $$C_{j}=\left\{\hat{b}\in {\hat{Y}}_{j}|\max_{b\in Y_{j}}\mathrm{IoU}\left(\hat{b},b\right)<\tau \right\}$$

where τ controls the filtering strength. This paper sets τ=0.45. The threshold defines a box-level verification query rather than an image-level active-learning request.

**Figure 3:**
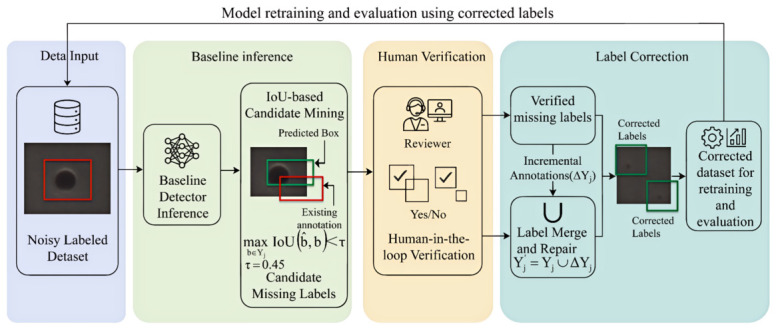
Workflow of active-verification-based label repair.

As shown in Figure 3, candidate missing labels are first obtained by running the baseline detector on data with noisy labels. Candidate boxes confirmed by humans as real defects are recorded as incremental annotations $\Delta Y_{j}$ and merged with the original annotation set:

(5) $$Y_{j}^{\prime }=Y_{j}\cup \Delta Y_{j}$$

Compared with standard human-in-the-loop active learning, the proposed stage differs in three aspects. First, the selection unit is an individual predicted box rather than a whole image. Second, the selection rule is not generic uncertainty but low IoU with all existing annotations, which directly targets the missing-label failure mode. Third, confirmed candidates are merged into the existing annotation set as additive increments, so the original labels are not replaced. This design makes the verification process inexpensive enough to repair both the training labels and the validation benchmark.

The checkpoint comparison is summarized in Table 2. The epoch sweep shows a quantity–quality trade-off. Epoch 10 nominates the largest number of boxes and recovers the largest number of confirmed defects, but it also has the lowest acceptance rate. Epochs 40 and 60 produce cleaner candidate pools but nominate fewer boxes. Epoch 22 is therefore used as a practical compromise between coverage and candidate purity, not as a claimed global optimum.

This procedure reduces the amount of manual work because reviewers only inspect model-nominated regions rather than relabeling all images. The same repair process is applied to both the training and validation sets.

### 2.4. Scale-Aware Prototype Consistency Feature Constraint

After label repair, the supervision quality is improved, but the target size distribution still exhibits a pronounced long-tailed pattern. Defects with normalized Area≤0.02 constitute the majority of samples. Under this distribution, the detector tends to develop a bias toward dominant small-size defects. In few-shot learning, classification is performed by computing distances to prototype representations of each class. This idea has been extended to few-shot object detection, anomaly detection, and anomaly localization.

Let $f_{i}^{t}$ denote the positive-sample region feature vector in training batch *t*. In implementation, $f_{i}^{t}$ is extracted from the small-object feature-pyramid level. The feature vector is taken at the grid cell containing the center of the corresponding ground-truth box. Because the resistor task is single-class defect detection, one defect prototype is used. For multi-class defect detection, the same formulation can be extended by maintaining one prototype per defect class.

The prototype is a batch-local mean, not an EMA or momentum-updated memory vector. It is recomputed from the positive samples in each batch and then L2-normalized:

(6) $$p^{t}=\frac{1}{N_{\mathrm{pos}}^{t}}\sum_{i\in \mathrm{pos}(t)}f_{i}^{t},\;{\hat{p}}^{t}=\frac{p^{t}}{∥p^{t}{∥}_{2}}.$$

Cosine similarity is adopted as the metric because it constrains the feature direction and is less sensitive to feature-norm variation in weak, low-contrast defects:

(7) $$\mathrm{Sim}\left(f_{i}^{t},{\hat{p}}^{t}\right)=\frac{f_{i}^{t}\cdot {\hat{p}}^{t}}{∥f_{i}^{t}{∥}_{2}{∥{\hat{p}}^{t}∥}_{2}}.$$

The prototype consistency constraint is imposed only on positive samples whose normalized area satisfies ${\mathrm{Area}}_{i}\leq \alpha$:

(8) $$L_{proto}=\frac{1}{N_{p}}\sum_{i=1}^{N_{p}}I\left({\mathrm{Area}}_{i}\leq \alpha \right)\left(1-\mathrm{Sim}(f_{i}^{t},{\hat{p}}^{t})\right).$$

Here, $N_{p}$ denotes the number of positive samples, I(·) is the indicator function, and α is the area-gating threshold set to α=0.02. The value α=0.02 corresponds to the empirical boundary between the dominant tiny/small groups and the rare normal-size group. The no-gate control shows that excluding the largest defects is not merely cosmetic. Otherwise, rare large defects can pull the prototype away from the dominant-scale direction and weaken the intended regularization.

The size-stratified Recall comparison is summarized in Table 3. The supplementary sweep further supports α=0.02 as the best overall operating point in that run. Larger thresholds or no-gate settings can improve individual size groups, but they do not improve the two overall metrics at the same time.

**Figure 4:**
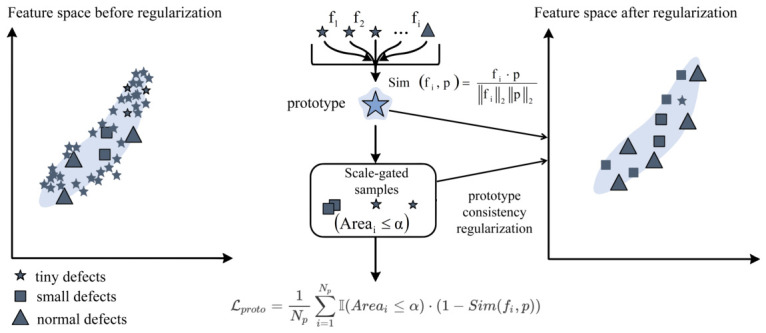
Illustration of the feature-space effect of the prototype consistency constraint.

This approach follows the principle in prototypical networks, and extends it with area-gated constraints informed by prototype distillation, prototype-guided feature learning, and noisy-label-aware defect representation. The feature-space intuition of this regularization is illustrated in Figure 4.

The total training loss is:

(9) $$L_{total}=L_{det}+\lambda L_{proto}$$

where λ balances the detection main task and feature regularization. The prototype term is used only when the training loss is computed. It is removed during inference and therefore does not change the deployed YOLO26s structure, parameter count, or runtime latency.

This staged design also has clear failure boundaries. If the prototype constraint is applied before label repair, the prototype can be biased by missing defects that are still treated as background. If the area gate is too narrow, too few positive samples contribute to a stable prototype within each batch. If the gate is removed or set too wide, the largest defects can dominate the mean prototype and reduce its scale selectivity. These limitations motivate the repair-first order and the selected α=0.02 setting.

## 3. Experiments and Result Analysis

The experiments are reported in the same order as the proposed pipeline. We first examine the effect of repairing missing labels, since this step changes both the training supervision and the validation benchmark. We then add the prototype term on the corrected labels and check whether the gain remains under the same split, input size, optimizer, training schedule, and random seeds.

### 3.1. Experimental Settings

Training was carried out on a Windows workstation using PyTorch v2.12.0.dev20260408+cu128 and the Ultralytics codebase (v8.4.57). The YOLO26s weights were initialized from the official implementation (https://github.com/ultralytics/ultralytics, accessed on 7 June 2026). In this configuration, the model has 9.47M parameters and 20.5 GFLOPs. All runs were completed on an NVIDIA GeForce RTX 5070 Ti Laptop GPU.

Unless a table states otherwise, the same data split, 1280×1280 input size, SGD optimizer, and 120-epoch schedule were used. The input size was not reduced because many labeled boxes occupy only a small area in the original images. The detector comparison and the ablation experiments therefore follow the same basic training recipe. Table 4 lists the main training parameters.

We built a task-specific dataset for resistor surface inspection. The validation split contains 58 images and 975 confirmed defect instances. Unlike public benchmark datasets, these images were collected from real production scenes, where labeling is affected by weak defect contrast and repetitive surface texture. For this reason, the original annotations contained missed boxes and a small number of inaccurate labels. If such labels are used directly, the detector is trained with noisy supervision [8], and the validation score may underestimate the true detection ability because correct predictions on missed defects can be counted as false positives.

During the active verification stage, candidate missing labels were mined, manually reviewed, and corrected separately for both the training and validation sets. As highlighted in active learning studies for industrial defect detection [21], the performance of deep learning-based methods is heavily dependent on the availability of accurately labeled datasets. Training-set correction was used to provide cleaner supervision for learning, whereas validation-set correction was used to obtain a less biased evaluation benchmark. In the raw validation labels, real defects missed by annotators may be counted as false positives once detected by the model; correcting these labels therefore avoids underestimating the detector. This correction step is part of the proposed framework rather than a separate preprocessing operation.

**Figure 5:**
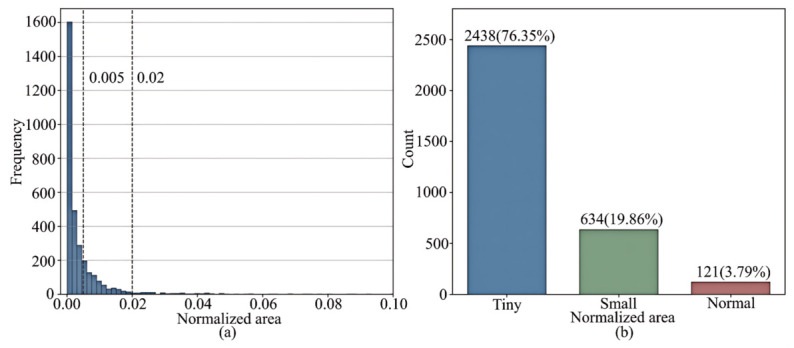
Statistical results of target size distribution in the dataset: (**a**) histogram of normalized defect area with the thresholds 0.005 and 0.02 marked; (**b**) counts and proportions of the Tiny, Small, and Normal groups after area-based partitioning.

The dataset is strongly skewed toward tiny defects. Among the 3193 analyzed boxes, 2438 boxes are tiny defects, accounting for 76.35%. Small defects account for 634 boxes, or 19.86%, while only 121 boxes, or 3.79%, belong to the normal-size group. The distribution is shown in Figure 5. This imbalance means that a detector can obtain a reasonable overall score while still performing poorly on the rare normal-size defects.

Figure 5 also explains why we report size-stratified Recall later. Since most boxes fall into the tiny-defect range, the overall mAP50 is mainly affected by the dominant group. It does not show whether the detector can still find the much rarer normal-size defects. Therefore, the later experiments separate the validation defects into tiny, small, and normal-size groups.

We report Precision, Recall, mAP50, and mAP50-95. Precision describes the reliability of predicted boxes, while Recall shows how many real defects are recovered. In this resistor inspection task, Recall is especially important because an undetected defect may pass into the next production stage, whereas an extra alarm can still be checked manually. mAP50 is used as the main overall metric at IoU = 0.5, and mAP50-95 is included to reflect stricter localization quality.

For the size-based evaluation, we divide the validation boxes by normalized area. Boxes with Area≤0.005 are assigned to the tiny group, boxes with 0.005<Area≤0.02 are assigned to the small group, and boxes with Area>0.02 are assigned to the normal-size group. This division is used because the dataset contains many tiny boxes but very few normal-size boxes. Each main experiment was repeated under five random seeds, and the reported values are mean ± standard deviation.

A balance-point metric is also reported in the horizontal comparison. It is computed from the precision–recall curve as the point where Precision and Recall are equal or the nearest interpolated point when an exact intersection is unavailable. This value provides an additional view of the trade-off between missed detections and false alarms.

### 3.2. Comprehensive Performance Comparison with Mainstream Detection Methods

Before the ablation study, we trained several commonly used detectors on the corrected labels. The comparison includes YOLOv8n [29] and YOLOv5s [30] as lightweight convolutional baselines, RT-DETR [31], Deformable DETR [32], and Swin Transformer [33] as Transformer-based baselines, and CenterNet [34] as an anchor-free baseline.

**Figure 6:**
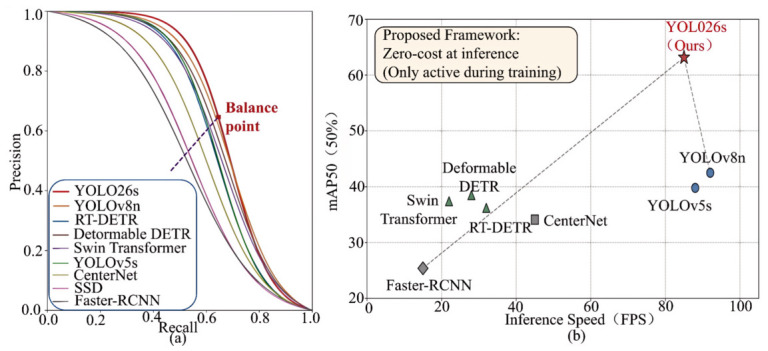
Comprehensive performance comparison with mainstream detection methods. (**a**) Precision–Recall curve comparison of different models; (**b**) Pareto-frontier distribution of different models in the two-dimensional space of accuracy and inference speed.

For this comparison, the corrected training set was used for every model. The input resolution, data split, and 120-epoch schedule were also kept the same as in Table 4. In this way, the table mainly reflects the effect of changing the detector architecture rather than changes in the training data or training length. The quantitative results are shown in Table 5 and Figure 6.

As shown in Table 5 and Figure 6a, replacing the model architecture alone does not lead to large performance gains on this dataset. Even YOLOv8n and Transformer-based models, after using clean labels (+AL), still produce mAP50 values in the range of 35–43%. Lightweight convolutional detectors perform slightly better than Transformer-based models for this task [12]; YOLOv8n reaches 42.50%, while Transformer-based methods are limited by the small data scale and sparse targets [14]. CenterNet shows weaker results for extremely tiny and weakly textured defects [13]. The proposed method, which adds a prototype consistency constraint on top of YOLO26s, reaches mAP50 of 63.15%, higher than all architecture-only baselines.

Figure 6b shows the accuracy–speed trade-off. Transformer-based models and CenterNet occupy the lower-left region with both low speed and low accuracy. YOLO-series baselines achieve FPS values above 80 but limited accuracy. The proposed YOLO26s (Ours) is located in the upper-right region: the prototype consistency constraint is used only during training, so the inference speed of YOLO26s (FPS > 80) is not affected. This suggests that, for this type of industrial task, combining data correction with feature regularization may be more practical than using heavier network architectures.

The remaining experiments fix YOLO26s as the baseline network. Since the task involves both missing-label noise and a long-tailed size distribution, changing the backbone would make it difficult to isolate the contributions of supervision repair and feature regularization. Three configurations are compared: (1) the Baseline model, trained and evaluated on the uncorrected raw dataset; (2) the AL model, retrained on labels corrected by active verification; (3) the AL+Proto model, which adds the prototype consistency constraint on top of the AL model.

### 3.3. Effectiveness and Parameter Sensitivity Analysis of Active-Verification-Based Label Repair

Active verification was tested as a label-repair step rather than as a new detector architecture. The baseline model first generated candidate boxes, and only candidates confirmed by human reviewers were added to the annotation files. In this industrial scenario, missing labels cause the training process to treat real defect regions as background, distorting the decision boundary. By addressing this problem at the data level through a “model nomination–human confirmation–label merging” loop, the network receives more accurate supervision.

In the training set, the detector nominated 945 candidate boxes, and 84 of them were accepted after manual checking. In the validation set, 1278 candidates were checked, and 116 were accepted. The acceptance rates were therefore 8.9% and 9.1%, respectively.

These numbers show why manual confirmation was kept in the loop. Most nominated boxes were not true missed defects, even though they had low overlap with the original labels. The candidates were generated from the Epoch-22 model with τ=0.45. At this stage, the model had already learned some defect locations, but it had not yet fully absorbed the missing-label regions as background.

**Figure 7:**
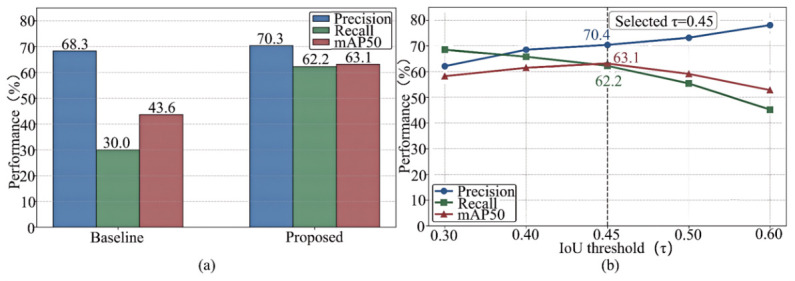
End-to-end overall effectiveness and parameter sensitivity analysis of the closed-loop framework. (**a**) Macroscopic performance comparison between the baseline model and the final framework; (**b**) sensitivity analysis of the matching threshold τ.

Table 6 is used as an end-to-end comparison between the raw-label setting and the final corrected setting. It is not treated as a strict one-factor ablation, because the validation labels are also corrected in the final setting. The matching-threshold sensitivity is therefore reported separately later in Table 7, while the fixed-benchmark ablation is discussed in the subsequent ablation subsection. The overall comparison is shown in Table 6 and Figure 7a.

In Table 6, the final setting gives a higher mAP50, from 43.66% to 63.15%, and a higher Recall, from 30.01% to 62.24%. The Recall change is the more important observation for this inspection task. Many of the repaired boxes correspond to defects that were visible but absent from the original labels. Once these boxes are included, detections on these regions are no longer counted as false positives, and the detector is also trained with fewer wrong background samples.

As observed in studies on learning from noisy labels [8], the quality of data labels is a concern because of the lack of high-quality labels in many real-world scenarios, and noisy labels severely degrade the generalization performance of deep neural networks. In this experiment, correcting the labels provides the detector with a more reliable optimization target by removing incorrect background supervision. Under this corrected supervision, the prototype constraint can further regularize the feature space and improve the detection of weak defect responses.

As shown in Table 7 and Figure 7b, as τ increases, Precision continues to rise, whereas Recall steadily declines. mAP50 exhibits an inverted-V trend and peaks at τ=0.45 with a value of 63.15%. If τ is too small, the system nominates many correct predictions with only slight box offsets, increasing manual workload. If τ is too large, independent tiny defects close to existing labels are directly ignored, weakening the mining capability. Considering both discovery cost and final accuracy, τ=0.45 is the optimal setting. In addition, the timing of baseline model extraction is also critical. This study selects the weights from the early training stage at Epoch 22 for missing-label mining. At this point, the model has acquired preliminary defect localization ability but has not yet deeply overfitted missing-label regions as background, thereby maximizing the candidate discovery potential of active verification [28].

### 3.4. Core Component Ablation and Size Generalization Analysis

**Figure 8:**
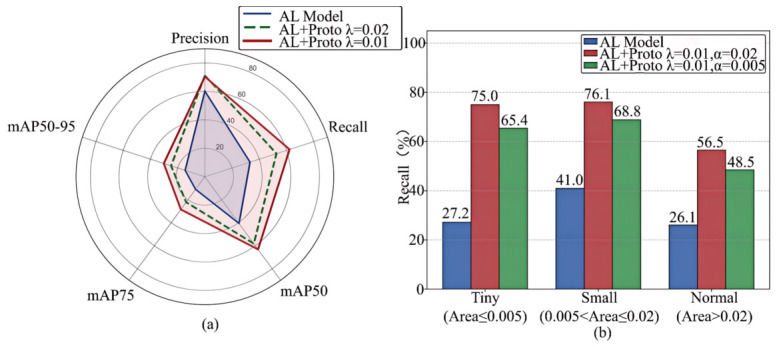
Multi-dimensional detection performance comparison under different model configurations and prototype constraint strengths: (**a**) radar-chart comparison of Precision, Recall, mAP50, mAP75, and mAP50-95 for the AL model and the two AL+Proto settings; (**b**) size-stratified Recall comparison for Tiny, Small, and Normal defects under the same settings.

After label repair, the training supervision is improved, but the long-tailed size distribution still causes the detector to favor dominant tiny defects. This section introduces the prototype consistency feature constraint and examines its effect from three perspectives: overall detection performance, size-stratified performance, and qualitative visualization. Progressive ablation experiments were conducted on the cleaned validation set. The results under different constraint strengths λ are shown in Table 8 and Figure 8.

Table 8 and Figure 8 show the progressive ablation results on the clean validation set. The Baseline trained on noisy labels has mAP50 of 28.14%. After active verification (AL model), mAP50 increases to 40.60%, a gain of 12.46%, but the long-tailed distribution still limits performance. Adding a prototype consistency constraint (λ=0.01) on top of AL raises mAP50 further to 63.20% (+22.60%) and Recall from 33.20% to 62.20% (+29.00%).

A self-training control experiment was also conducted to check whether manual review can be skipped. This experiment uses the same candidate mining process as active verification, with the same baseline weights and IoU threshold τ=0.45, but all candidate boxes are directly merged as pseudo-labels without human confirmation. As shown in Table 8, the self-training result shows the risk of accepting all candidates without review. Although Recall appears high (66.90%), Precision collapses to 5.59%, indicating that the model responds to nearly all regions after being contaminated by false-positive pseudo-labels. Since only about 9% of reviewed candidates were accepted in the active-verification step, directly merging all candidates introduces more noise than useful supervision. In this dataset, the result indicates that manual review is still needed before pseudo-labels are merged.

The prototype term is added only after the labels have been repaired. It uses positive samples to form a defect prototype in the feature space and then penalizes large angular deviations from this prototype. In our runs, this regularization mainly changes the behavior of weak defects: their features are less likely to drift toward the background side after training.

The weight λ controls how strongly the prototype term affects the detector during training. When λ was increased to 0.02, Precision rose to 71.20%, but Recall dropped to 52.60%, and mAP50 decreased to 58.20%. This behavior suggests that the detector became more conservative. Such a result is not suitable for our inspection target, because missing a real defect is more costly than producing an additional candidate alarm. A likely explanation is that the stronger prototype term pulls positive features too tightly toward the batch prototype. Defects with weak contrast, unusual shape, or larger area may then be treated as less typical positives and become easier to miss.

The setting λ=0.01 was more suitable in the five-seed experiments. It improved Recall without making the positive features overly compact. This result also shows that the prototype term should not be treated as a stronger-is-always-better constraint. With λ=0.01, the mAP50 standard deviation decreased from ±0.55% to ±0.38%, indicating more stable training on the corrected labels. Based on this setting, we next examine Recall for tiny, small, and normal-size defects separately.

The 12.46% mAP50 increase from Baseline to AL should be read together with the label-repair process. The corrected validation set is not made easier by removing difficult samples. Instead, it adds boxes for real defects that were missing in the raw annotations. Before correction, a prediction on one of these regions could be counted as a false positive even when the image evidence supported the detection. After correction, both training and evaluation are closer to the actual inspection target.

For the 22.60% mAP50 improvement from AL to AL+Proto, the AL model (40.60%) is not inherited from intermediate weights but is trained from scratch on the same corrected dataset using the same hyperparameters and random seeds across five repetitions. The AL model therefore serves as a strict control for AL+Proto with the prototype loss removed (λ=0). Under this controlled setting, the difference is mainly associated with the addition of the prototype term and its regularization effect on the feature space.

To further verify the mechanism of “correcting feature bias,” this study divides validation-set defects into three categories according to the normalized target area: tiny defects (Area≤0.005), small defects (0.005<Area≤0.02), and normal-size defects (Area>0.02). The corresponding stratified Recall results are shown in Table 9.

**Figure 9:**
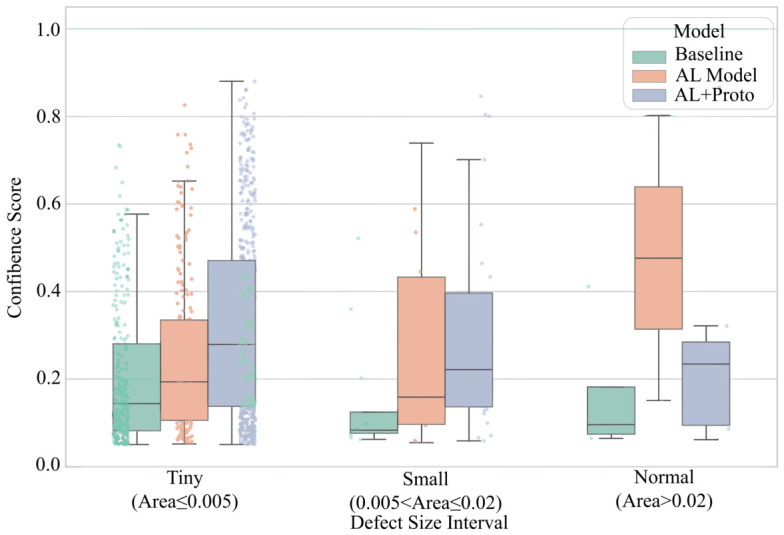
Prediction confidence distributions across different defect size intervals. Compared with the baseline model and the pure AL model, the AL+Proto method compresses the prediction variance of non-dominant large-size defects, namely the Normal class, indicating improved cross-scale feature stability.

**Figure 10:**
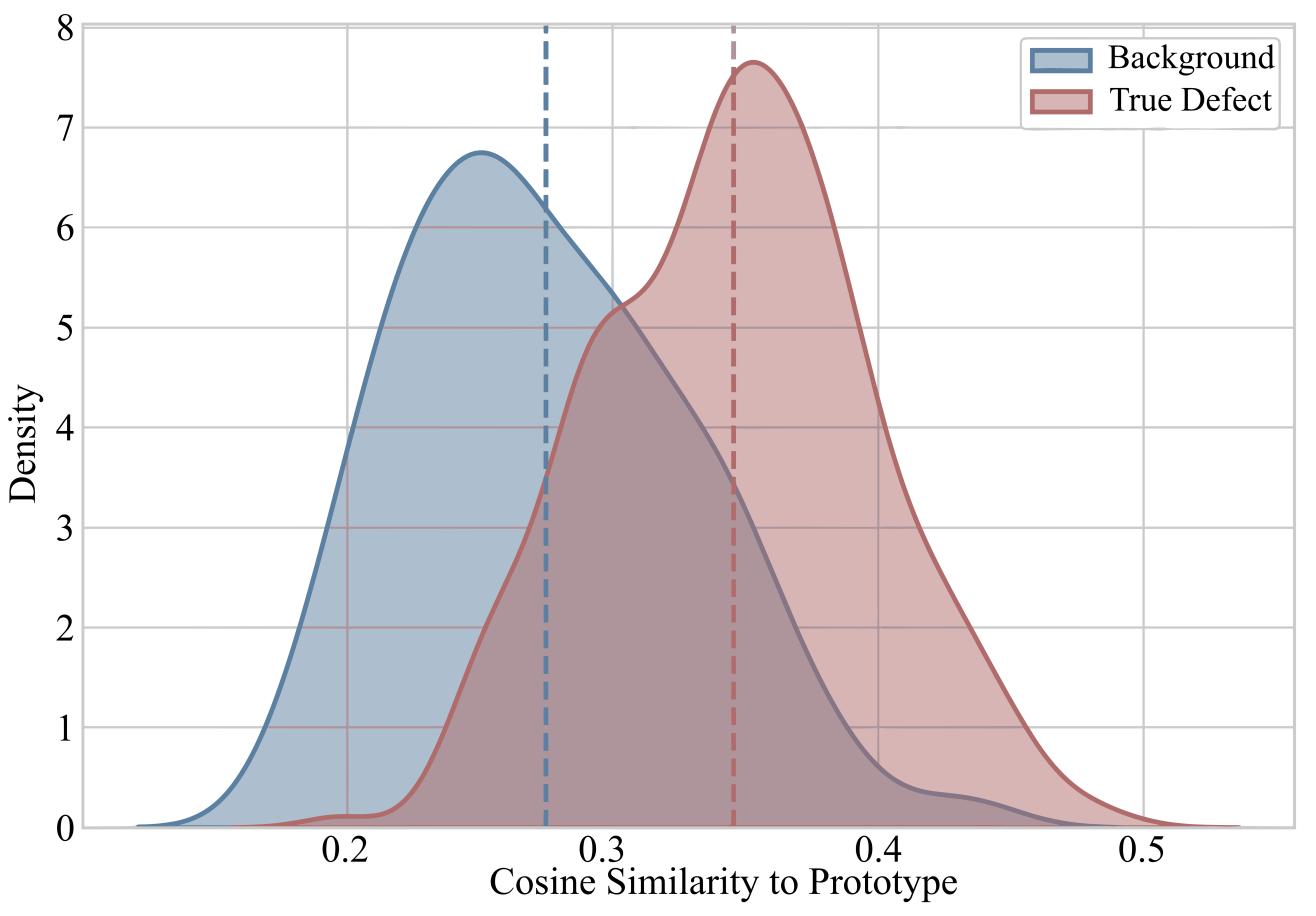
Kernel density estimation distribution of cosine similarity between ROI features and the defect prototype. The main density peaks of real defect features (red) and background features (blue) exhibit clear inter-class separation, indicating the effective discriminative clustering ability of the proposed prototype mechanism.

In the experimental design of Table 9, α=0.02 is selected as a relaxed boundary covering approximately 96% of defect samples, whereas α=0.005 covers only the most dominant tiny-defect group. These two settings are used to examine how area gating controls the effective range of feature regularization. The pure AL model shows low Recall for the Normal category (26.09%), indicating that the network is biased toward the dominant tiny samples. After introducing the prototype constraint with α=0.02, the Recall of both Tiny and Small categories increases, and the Recall of Normal defects rises to 56.52% with narrowed variance. This result indicates that the constraint acts as a cross-scale feature smoothing mechanism and reduces the size bias caused by the long-tailed distribution. At the prediction level, as shown by the boxplot in Figure 9, AL+Proto compresses the prediction variance of non-dominant large-size defects (Normal class) and increases the median confidence. The kernel density estimation in Figure 10 shows that real defect features and background features have visible separation along the prototype-similarity dimension, which is consistent with the intended clustering effect of the prototype vector.

**Figure 11:**
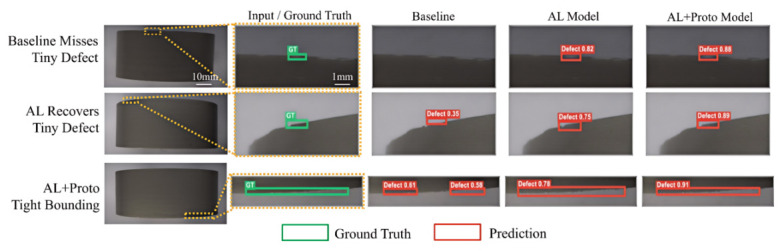
Visual comparison of detection results from different models.

The qualitative visualization in Figure 11 is consistent with the quantitative results. In tiny and weak-texture scenarios, the AL+Proto model recovers targets missed by the Baseline and assigns higher confidence scores. In larger-size scenarios, AL+Proto outputs more complete bounding boxes compared with the fragmented local responses of the Baseline. Together, these observations support the conclusion that the proposed framework improves detection across different defect sizes.

## 4. Conclusions

This paper presented a missing-annotation-aware method for tiny resistor surface defect detection. The method first uses active verification to identify and confirm suspected missing labels, and then applies a scale-aware prototype consistency constraint during training. In this way, the detector is trained with more complete supervision while its feature space is regularized for the long-tailed defect-size distribution. On the cleaned validation benchmark, the proposed framework improves mAP50 from 43.66% to 63.15% and Recall from 30.01% to 62.24%. Since noisy labels severely degrade the generalization performance of deep neural networks [8], the experiment confirms that missing annotations are an important source of performance loss in this task. After label repair, the prototype constraint further improves the detection of weak and non-dominant-size defects without changing the inference structure of YOLO26s.

The present study is still based on one resistor dataset. Future work should first test the same procedure on public defect datasets, such as NEU-DET and GC10-DET. The manual checking step also needs to be made cheaper, for example by ranking candidate boxes before review or grouping similar candidates together. Another useful test is to apply the method to wafer inspection and metal casting, where weak defects and missing labels may occur at the same time.

## Figures and Tables

**Table 1:** Dataset composition after annotation repair.

Item	Value
Cleaned validation images	58
Confirmed validation defect instances	975
Boxes used for size-distribution analysis	3193
Tiny defects	2438 (76.35%)
Small defects	634 (19.86%)
Normal-size defects	121 (3.79%)
Separate held-out test set	Not available in the current production dataset

**Table 2:** Effect of the missing-label mining checkpoint on candidate quantity and confirmation rate.

Epoch	Candidate Boxes	Confirmed Boxes	Acceptance Rate
10	19,478	9630	49.44%
22	13,731	8014	58.36%
40	12,666	8131	64.20%
60	12,666	8131	64.20%

**Table 3:** Size-stratified Recall used to justify the area gate in the revised manuscript.

Configuration	Tiny Recall	Small Recall	Normal Recall
AL without prototype constraint	27.23%	40.96%	26.09%
AL+Proto (λ=0.01, α=0.02)	75.00%	76.06%	56.52%
AL+Proto (λ=0.01, α=0.005)	65.40%	68.80%	48.50%

**Table 4:** Experimental environment and main training parameter settings.

Parameter	Setting	Description
Baseline architecture	YOLO26s	Balances lightweight design and feature extraction capability
Model scale	9.47M Params, 20.5 GFLOPs	Satisfies lightweight industrial deployment requirements
Deep learning framework	PyTorch + Ultralytics	Used for end-to-end model training and inference
Input resolution	1280×1280	High-resolution setting for preserving tiny defect features
Batch size	4	Balanced configuration under GPU memory constraints
Epochs/Patience	120/60	Early stopping is used to prevent overfitting
Optimizer	SGD	Momentum = 0.937, weight decay = 0.0005
Initial learning rate	0.01	Standard initial learning rate for training
Loss weights	Box = 7.5, Cls = 0.5, DFL = 1.5	Default detection-framework weighting for fair baselines
Test confidence threshold	0.05	Engineering setting oriented toward high recall

**Table 5:** Performance comparison with mainstream detection methods based on the high-quality corrected dataset. All models were trained for 120 epochs using the same corrected training set and input resolution.

Model Architecture	Method Type	mAP50 (%)	FPS	BP Recall	BP Prec.
**Proposed (YOLO26s + AL+Proto)**	**CNN + constraint**	**63.15**	**85**	**0.622**	**0.704**
YOLOv8n (+AL)	CNN (lightweight)	42.50	102	0.354	0.612
YOLO26s (+AL)	CNN (lightweight)	40.63	85	0.332	0.602
YOLOv5s (+AL)	CNN (lightweight)	39.80	91	0.315	0.585
Deformable DETR (+AL)	Transformer	38.50	18	0.308	0.574
Swin Transformer (+AL)	Transformer	37.40	12	0.295	0.561
RT-DETR (+AL)	Transformer	36.20	32	0.288	0.542
CenterNet (+AL)	Anchor-free	34.10	45	0.272	0.515

**Table 6:** Cross-benchmark end-to-end performance comparison before and after closed-loop framework deployment based on five independent experiments.

Model Stage	Evaluation Set	Precision (%)	Recall (%)	mAP50 (%)
Initial Baseline	Raw noisy validation set	68.32±0.65	30.01±0.82	43.66±0.74
Final (AL+Proto)	Cleaned validation set	70.38±0.48	62.24±0.55	63.15±0.42

**Table 7:** Sensitivity analysis of the matching threshold τ.

IoU Threshold (τ)	Precision	Recall	mAP50	Phenomenon Analysis
0.30	0.621	0.685	0.582	Too low; slight offsets treated as missing labels, high review cost
0.40	0.685	0.658	0.615	Close to balance but insufficient boundary tolerance
**0.45**	**0.704**	**0.622**	**0.631**	**Best balance between precision and recall**
0.50	0.732	0.554	0.591	Some real missing labels regarded as already annotated
0.60	0.781	0.452	0.528	Too high; active discovery clearly weakened

**Table 8:** Multi-dimensional detection performance comparison under different prototype constraint strengths based on the clean validation set, reported as mean ± standard deviation.

Configuration	Prec. (%)	Recall (%)	mAP50 (%)	mAP75 (%)	mAP50-95 (%)
Baseline	54.32±0.62	18.42±0.75	28.14±0.68	7.62±0.45	9.85±0.50
Self-training ^†^	5.59	66.90	5.88	—	2.46
AL model	60.20±0.52	33.20±0.68	40.60±0.55	11.00±0.50	14.50±0.45
AL+Proto (λ=0.01)	70.40±0.35	62.20±0.45	63.20±0.38	28.50±0.35	30.20±0.40
AL+Proto (λ=0.02)	71.20±0.28	52.60±0.40	58.20±0.32	22.20±0.40	24.80±0.35

^†^ Self-training is a single-run result. It adopts the same candidate mining process as active verification (τ=0.45), but skips manual review and directly merges all candidate boxes as pseudo-labels.

**Table 9:** Size-stratified generalization analysis (mean ± standard deviation, IoU = 0.5).

Model Configuration	Tiny Recall (Area ≤ 0.005, *N* = 764)	Small Recall (0.005 < Area ≤ 0.02, *N* = 188)	Normal Recall (Area > 0.02, *N* = 23)
AL model without constraint	27.23±0.85	40.96±0.65	26.09±2.15
AL+Proto (λ=0.01, α=0.02)	75.00±0.48	76.06±0.35	56.52±0.80
AL+Proto (λ=0.01, α=0.005)	65.40±0.60	68.80±0.42	48.50±1.10

## Data Availability

The data presented in this study are not publicly available due to industrial confidentiality restrictions. Data may be available from the corresponding authors upon reasonable request and with permission from the industrial partner.
